# Pre-Menopausal Women With Breast Cancers Having High AR/ER Ratios in the Context of Higher Circulating Testosterone Tend to Have Poorer Outcomes

**DOI:** 10.3389/fendo.2021.679756

**Published:** 2021-06-21

**Authors:** Savitha Rajarajan, Aruna Korlimarla, Annie Alexander, C. E. Anupama, Rakesh Ramesh, B. S. Srinath, T. S. Sridhar, Jyothi S. Prabhu

**Affiliations:** ^1^ Division of Molecular Medicine, St. John’s Research Institute, Bangalore, India; ^2^ Centre for Doctoral Studies, Manipal Academy of Higher Education (MAHE), Manipal, India; ^3^ Department of Research, Sri Shankara Cancer Hospital and Research Centre, Bangalore, India; ^4^ Department of Surgical Oncology, St. John’s Medical College and Hospital, Bangalore, India; ^5^ Department of Surgery, Sri Shankara Cancer Hospital and Research Centre, Bangalore, India

**Keywords:** breast cancer, androgen receptor, AR/ER ratio, pre-menopausal, testosterone

## Abstract

**Purpose:**

Women with breast tumors with higher expression of AR are in general known to have better survival outcomes while a high AR/ER ratio is associated with poor outcomes in hormone receptor positive breast cancers mostly in post menopausal women. We have evaluated the AR/ER ratio in the context of circulating androgens specifically in patients younger than 50 years most of whom are pre-menopausal and hence have a high estrogenic hormonal milieu.

**Methods:**

Tumor samples from patients 50 years or younger at first diagnosis were chosen from a larger cohort of 270 patients with median follow-up of 72 months. Expression levels of ER and AR proteins were detected by immunohistochemistry (IHC) and the transcript levels by quantitative PCR. Ciculating levels of total testosterone were estimated from serum samples. A ratio of AR/ER was derived using the transcript levels, and tumors were dichotomized into high and low ratio groups based on the third quartile value. Survival and the prognostic significance of the ratio was compared between the low and high ratio groups in all tumors and also within ER positive tumors. Results were further validated in external datasets (TCGA and METABRIC).

**Results:**

Eighty-eight (32%) patients were ≤50 years, with 22 having high AR/ER ratio calculated using the transcript levels. Circulating levels of total testosterone were higher in women whose tumors had a high AR/ER ratio (p = 0.02). Tumors with high AR/ER ratio had significantly poorer disease-free survival than those with low AR/ER ratio [HR-2.6 (95% CI-1.02–6.59) p = 0.04]. Evaluation of tumors with high AR/ER ratio within ER positive tumors alone reconfirmed the prognostic relevance of the high AR/ER ratio with a significant hazard ratio of 4.6 (95% CI-1.35–15.37, p = 0.01). Similar trends were observed in the TCGA and METABRIC dataset.

**Conclusion:**

Our data in pre-menopausal women with breast cancer suggest that it is not merely the presence or absence of AR expression but the relative activity of ER, as well as the hormonal milieu of the patient that determine clinical outcomes, indicating that both context and interactions ultimately influence tumor behavior.

## Introduction

Breast cancer in the young is more commonly associated with aggressive features and poorer clinical outcomes when compared to that of an older age group (1). Although the incidence of breast cancer in women ≤50 years is limited to less than a third in most clinical series, proportions seem to vary in different ethnic populations (2, 3). Hormonal risk factors are different in this age group, and younger women tend to have more hormone receptor negative breast cancers with adverse prognostic features (4). Recent studies which have characterized genomic and transcriptomic profile of the breast cancers in the young and pre-menopausal women have shown them as a unique etiologic and biological entity (5).

Circulating androgens are detected during all ages in adult women and hence thought to have biological roles (6). Multiple studies both in pre- and post-menopausal women have reported a significant positive association between higher levels of circulating androgens and the risk of developing breast cancer (7–10). Contrary to the action of androgens which mediate their effects through androgen receptor (AR), expression of AR has been shown to be a favorable prognostic indicator in breast cancer. Women with estrogen receptor (ER) positive, AR positive (ER+AR+) tumors are known to have better survival and more favorable clinicopathological features, like negative lymph node metastasis and lower tumor grade than women whose tumors are negative for AR (11).

The clinical and biological significance of AR expression in breast cancer is not straight forward due to variations in both the levels of AR as well as the intrinsic differences among the multiple subtypes of breast cancer. AR is known to control tumor growth in ER positive tumors and stimulate disease progression in the absence of ER (12). *In vitro* studies have demonstrated that AR might decrease ER transcriptional activity probably by competing to the same binding sites as ER in breast tumors (13). However, Cochrane et al. were the first to report high levels of AR could be associated with a worse prognosis with tamoxifen resistance and defined the relationship between AR and ER expression as AR/ER ratio in ER positive tumors to display the dynamic interplay between the two receptors (14). Multiple other studies since then have evaluated the utility of AR/ER ratio and shown higher ratios were associated with unfavorable features and poor prognosis in breast cancer (15–18). Most of them have however, focused on ER positive, HER2 negative subgroup of breast tumors in elderly women (median age >60 years) in predominant Caucasian women. Breast cancer in the south Asian population is seen to arise at least a decade earlier with half of the women less than 50 years of age at first diagnosis (19, 20). In this study, we have investigated the role of AR by evaluating the AR/ER ratio specifically in patients younger than 50 years of age and who are likely to have a dominant estrogenic environment and the role of this particular hormonal environment on tumor progression.

## Methods

### Cohort Details

Tumor samples were chosen from a retrospective cohort of 270 women with primary breast cancer including five women with bilateral tumors. These samples were collected as part of an observational longitudinal study from two tertiary cancer care hospitals in Bangalore, India between 2008 and 2013, and these women were followed-up for up to 9 years, with a total loss to follow-up of less than 5% and a median follow-up duration of more than 72 months. The study was approved by the ethical committee of both institutions, and informed consent was obtained from all the patients to use their tissue and blood sample for research. Information on clinical variables like age, grade, tumor size, lymph node status, stage of the disease with ER, progesterone receptor (PgR), and HER2 was obtained from their clinical records. Treatment information was obtained from clinical records of patients during follow-up. Endocrine therapy was recorded as tamoxifen or aromatase inhibitor, and chemotherapy regimens were noted for intake of anthracyclines or taxanes. Information on trastuzumab was recorded in HER2 positive patients whenever received. Formalin fixed paraffin embedded (FFPE) blocks from tumor tissue having more than 50% of the area of representative tumor were selected for the study.

### Immunohistochemistry of AR

Immunohistochemistry for AR was done on each of the tumor sections as per standard protocol using the Ventana Benchmark^XT^ staining system (Ventana Medical Systems, Tucson, AZ, USA). Briefly, 5 μm thick sections were fixed in hot air oven at 60°C for 60 min and loaded on to an IHC staining machine. De-paraffinization was performed using EZ Prep solution (Proprietary-Ventana reagent), and antigen retrieval was done using Cell Conditioning solution 1 (CC1) for 60 min. Primary antibody for AR (Clone AR 441, DAKO, dilution at 1:75) was added manually and incubated for 32 min at room temperature. Optiview DAB Detection Kit (Ventana Medical Systems) was used to visualize the signal, using DAB (3–3′diaminobenzidine) as the chromogen. Further, the sections were automatically counterstained with hematoxylin II (Ventana Medical Systems) for 12 min. The slides were removed from the autostainer, washed in de-ionized water, dehydrated in graded ethanol, cleared in xylene, and examined by microscopy. Appropriate positive and negative controls were run for each batch. Two pathologists scored the staining for AR protein independently and arrived at a final score. Nuclear staining in more >1% of tumor cells was considered as positive.

### RNA Extraction, cDNA Conversion, and Real Time PCR

Total RNA was extracted using the Tri Reagent protocol according to manufacturer’s instructions (Sigma Aldrich # T9424) from two 20 µm sections from the selected tumor block. Briefly, tumor block was deparaffinized using heat, and then subjected to overnight digestion using proteinase K (Qiagen #19133). Quantitation of the RNA was done using the Qubit RNA BR (Broad-Range) Assay Kit (Invitrogen # Q10210) on a Qubit 2.0 Fluorometer (Invitrogen #Q32866). Then 500 ng of total RNA was reverse transcribed to cDNA using high capacity cDNA conversion kit from Thermofisher scientific (Cat # 4322171) as per manufacturer’s instruction.

Primers were designed for *AR* and *ESR1* genes using primer 3 plus software and further validated on ensemble genome browser, NCBI blast and UCSC genome browser. The primers were synthesized by Juniper Life Sciences, Bangalore, India. The details of the primer sequences are given in the **Supplementary Table 1**. For quantitative real time PCR (qPCR), 5 ng of cDNA template was used per reaction and performed in duplicate using SYBR^®^ Green on the LightCycler^®^ 480 II (Roche Diagnostics). Pre-incubation and initial denaturation of the template cDNA were performed at 95°C for 10 min, followed by amplification for 45 cycles at 95°C for 15 s and 60°C for 1 min. Cycles of threshold (Ct) values for the test genes were normalized to the mean Ct values of the three reference genes—*ACTB, RPLP0*, and *PUM1* for each tumor sample which was normalized for varying abundance of transcripts. Relative normalized expression of test genes was calculated by ΔCT method. The methods used for nucleic acid extraction, quantitative PCR (qPCR), and selection of housekeeping genes (HKGs) and the quality control criteria for inclusion of samples in the analysis have been described in detail in our previous publication (21).

### Estimation of Total Testosterone

The estimation of total testosterone in serum samples collected prior to surgery or following surgery of 169 breast cancer patients was done by a chemiluminescence based immunoassay method using the Abbott Architect ci8200 (Integrated) & i2000 (Immunoassay) instrument. In brief, the serum sample with a minimum volume of 300 µl was loaded onto the instrument. The sample was then transferred into multiple compartments where it is mixed, incubated, and washed. In the subsequent steps, the conjugate, pre-trigger and trigger solutions were added. The chemiluminescence emission was measured to determine the quantity of total testosterone in the serum sample. The result was calculated using a four parametric logistic curve fit data reduction method to generate a calibration curve.

### Statistical Methods

Descriptive analysis was done to evaluate the cohort characteristics and distribution of the high and low AR/ER ratio groups. Difference in the clinical variables between high and low ratio groups was tested by independent Student’s t-test or Mann–Whitney U test for continuous variables, and chi-square test was done for categorical variables. Concordance between the AR transcript and protein was estimated by receiver operating characteristic (ROC) curve analysis. Kaplan–Meier survival curves and log rank tests were used to compare the disease-free and breast cancer specific survival between the high and low AR/ER ratio groups. Disease free survival (DFS) and breast cancer specific survival (BCSS) were calculated as the time from the date of first diagnosis to the time when a local or distant recurrence occurred and death due to disease, respectively. Patients with no event or had death due to non-breast cancer related causes were right censored. The prognostic importance of high AR/ER ratio in comparison to other clinicopathological characteristics was validated by both univariate and multivariate cox-proportional hazard analyses. All tests were two tailed, and P-value <0.05 was considered statistically significant. All statistical analyses were done on statistical software XLSTAT version 2019.4.2 and SPSS software version 20 (Chicago, IL).

## Results

### Patient Characteristics

A total of 270 patients were included in the study with a median age at first diagnosis of 56.2 years. Nearly 60% of the tumors were associated with spread to the regional lymph-nodes and half of women were at clinical stage 2 and a third stage 3. Less than 10% of the tumors were grade 1 with approximately half being grade 2; 68% were estrogen receptor positive, and 19% were HER2 positive. Clinical variables are shown in **Table 1**.

**Table 1:** Clinicopathological features of all patients and patients ≤50 years in our cohort.

Clinicopathological characteristics		All patients(N = 270)	Patients≤50 years(N = 88)
		N (%)	N (%)
Age	Median	56	43
T size	Median	3	3
	T1	72 (27)	22 (25)
	T2	160 (59)	50 (57)
	T3	29 (11)	12 (14)
	Unknown	9 (3)	4 (4)
Lymph Node	Positive	157 (59)	53 (60)
	Negative	104 (38)	34 (39)
	Unknown	9 (3)	1(1)
Stage	I	41 (15)	15 (17)
	II	133 (49)	43 (49)
	III	86 (32)	23 (26)
	IV	10 (4)	7 (8)
Grade	I	19 (7)	5 (5)
	II	131 (48)	42 (48)
	III	117 (43)	40 (46)
	Not available	3 (1)	1 (1)
Menopausal status	Pre	75 (28)	70 (80)
	Post	195 (72)	18 (20)
Estrogen Receptor	Positive	186 (68)	54 (61)
	Negative	89 (32)	34 (39)
Progesterone Receptor	Positive	172 (63)	55 (63)
	Negative	103 (37)	33 (37)
HER2	Positive	53 (19)	20 (23)
	Negative	192 (70)	58 (66)
	Equivocal	30 (11)	10 (11)

Most of the patients (>95%) were treated with stage appropriate endocrine and chemotherapy as standard of care except those with stage IV disease who died due to disease before completion of therapy. Of ER positive patients 93% (50/54) received endocrine therapy and received stage appropriate chemotherapy as well. Similarly, in ER negative patients, more than 90% of patients received stage appropriate chemotherapy and one had defaulted. Only 15% (3/20) of the HER2 positive patients received trastuzumab while 95% of them received anthracycline and taxane based regimens as intensive chemotherapy.

Among the 270 patients, 88 women were less than or equal to 50 years at first diagnosis, and the median age of this subset was 43.1 years. In this subset, 60% of the tumors were lymph node positive, and nearly half of the tumors belonged to stage II. Ninety-five percent of the tumors were equally distributed between grades II and III. Sixty percent of the tumors were ER positive. Eighty percent of these patients were pre-menopausal (70/88), and the remainder had been diagnosed with breast cancer on average within 3 years of menopause. No significant difference in any of the clinical characteristics was observed when this subgroup was compared to the entire cohort (**Table 1**).

### Expression of AR Protein, Transcript, and Concordance With ER Protein

Immunohistochemistry for AR could be successfully evaluated in 189 of the 275 tumors. Eighty six tumors were not evaluated either due to insufficient tissue or tumor content. An additional 12 tumors were excluded due to poor tissue preservation, and hence the final evaluation included only 177 tumors from 173 women. There were 59/173 women (34%) less than 50 years, and 114/173 women (66%) were >50 years of age.

Overall, 66/177 (37%) tumors had nuclear staining for AR. There was no difference in the distribution of AR protein by age groups (34% (20/59) in ≤50 and 39% (46/118) in >50 years age group). Of the 177 tumors, 120 were ER positive by IHC; 53/120 of these ER positive tumors were AR positive as well, and this proportion did not differ between the age groups [42% (16/38) *vs* 45% (37/82) in ≤50 years *vs >*50 years respectively]. Overall, only 30% (53/177) of the tumors were dual positive for both ER and AR. Of AR positive tumors, 80% were ER positive in all samples, and similar results were seen in both age groups.

Tumors which were positive for AR protein expression had significantly high levels of AR transcripts than AR negative (p = 0.003). Tumors in the >50 years group had higher levels of AR transcripts when compared to ≤50 age group (p = 0.021). ROC analysis showed only moderate concordance (AUC of 0.63, p = 0.07) between the transcript and the protein across all tumors. No difference in this concordance was observed when stratified by age groups.

### AR/ER Ratio by Transcript Levels

The *AR* and *ESR1* transcript levels were evaluated by real time PCR on all the 275 breast tumor samples. A significant positive correlation was observed between the *AR* and *ESR1* transcript levels (Pearson’s r = 0.43, p < 0.0001). Relative normalized units of *AR* and *ESR1* transcripts were used to calculate the AR/ER transcript ratio which ranged from 0.65 to 5.53. In the tumors of women ≤50 years of age, the ratio ranged from 0.65 to 3.53 with a median value of 1.46 and third quartile value of 1.75. In tumors from women over 50 years of age, the ratio ranged from 0.74 to 5.53 and had a median of 1.31 and third quartile of 1.57. Though the level of AR transcript was higher in >50 years group, the AR/ER ratio was significantly higher in tumors ≤50 years than in tumors >50 years (p = 0.005).

We further divided the tumors from women ≤50 years of age into high and low ratio groups based on the third quartile cut-off of 1.75, and 22/88 had high AR/ER ratio in this subset. Comparison of clinical characters between the high and low ratio groups showed higher preponderance of ER negative tumors in the high ratio group (68%, p = 0.001), and no significant differences were observed in other features like stage, grade, lymph node status, and tumor size as shown in **Table 2**.

**Table 2:** Comparison of clinical variables between high and low AR/ER ratio groups in our cohort in the patients ≤50 years.

Clinicopathological characteristics		High AR/ER ratio(N = 22)	Low AR/ER ratio(N = 66)	p-value
		N (%)	N (%)	
Age	Median	43	43	
T size	Median	3.25	3	0.92
	T1	6 (27)	16 (24)	0.57
	T2	10 (46)	40 (61)	
	T3	4 (18)	8 (12)	
	Unknown	2 (9)	2 (3)	
Lymph Node	Positive	14(64)	39 (60)	
	Negative	8 (36)	26 (40)	0.715
Stage	I	5 (22)	10 (15)	0.807
	II	9 (40)	34 (51)	
	III	6 (27)	17 (25)	
	IV	2 (9)	5 (7)	
Grade	I	0	5 (7)	0.18
	II	9 (40)	33 (51)	
	III	13 (60)	27 (41)	
	Not available		1 (1)	
Estrogen Receptor	Positive	7 (32)	47 (71)	0.001*
	Negative	15 (68)	19 (29)	
Progesterone Receptor	Positive	7 (32)	48 (73)	0.001*
	Negative	15 (68)	18 (27)	
HER2	Positive	6 (27)	14 (21)	0.421
	Negative	15 (68)	43 (65)	
	Equivocal	1 (4)	9 (14)	

*p-value <0.05, statistically significant.

### Patients With High AR/ER Ratio Had Poor Survival in ≤50 Years Age Group

We first examined the prognostic ability of ER and AR independently both at protein and transcript levels in women ≤50 years by Kaplan–Meier survival analysis. No significant difference in survival was seen for ER protein (ER positive *vs* negative, mean survival 76.63 *vs* 76.98 months, log rank test p = 0.65) and ER transcript at mean cut-off (high *vs* low, mean survival time 81.76 *vs* 74.68 months, log rank test p = 0.75). Similarly, no difference in survival was seen with AR protein (AR positive *vs* negative, 75 *vs* 81.98 months, log rank test p = 0.18) or its transcript levels at mean cut-off (AR high *vs* low, 78.4 *vs* 76.7 months, log rank test p = 0.55).

**Figure 1:**
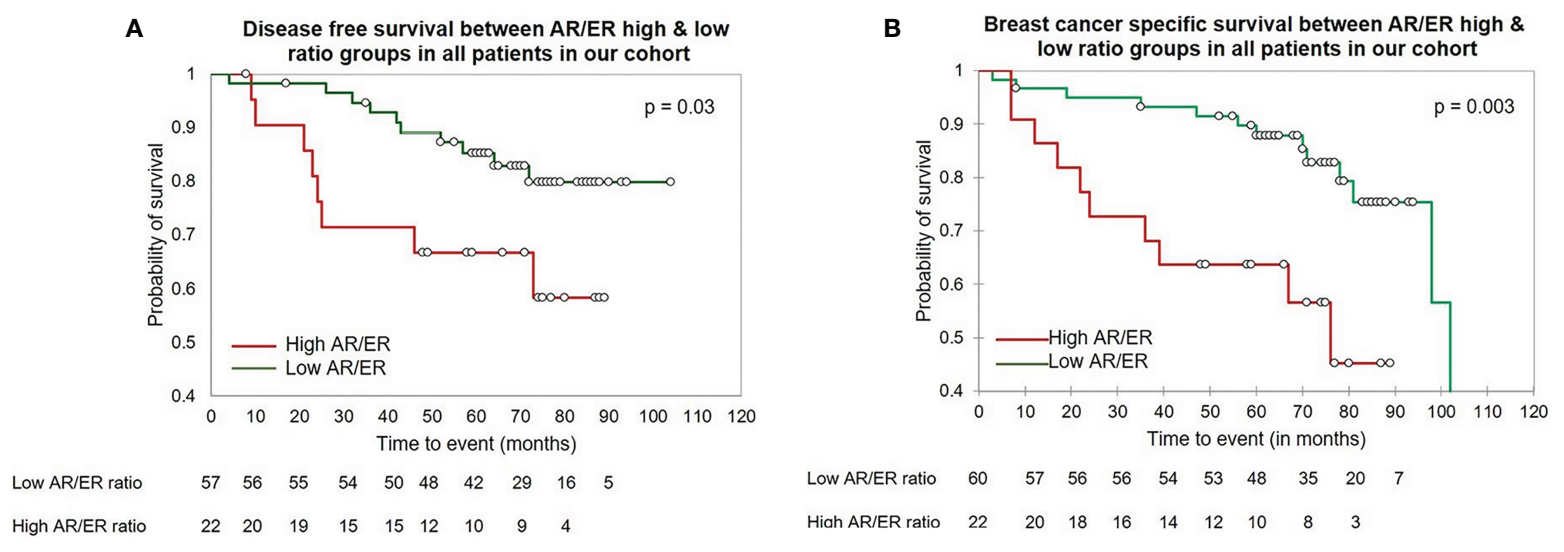
The Kaplan–Meier survival analysis in our cohort in the patients ≤50 years of age **(A)** The disease-free survival between the high *vs* low AR/ER ratio groups. **(B)** The breast cancer specific survival between the high *vs* low AR/ER ratio groups.

Next, we examined the clinical significance of a higher AR/ER ratio in the age group of patients ≤50 years group by Kaplan–Meier survival analysis. As seen in the **Figures 1A, B**, both DFS and BCCS were significantly lesser in the high ratio group in comparison to low ratio group (mean survival time 64.9 *vs* 83.4 months, log rank test p = 0.01 for DFS and 56.99 *vs* 89.65 months, log rank test p = 0.003 for BCSS). We did not observe this difference in the survival in the >50 years of age group, though trends were indicative of better survival for low ratio group (mean survival time 66.9 *vs* 81.13 months, log rank test p = 0.1 for DFS).

**Figure 2:**
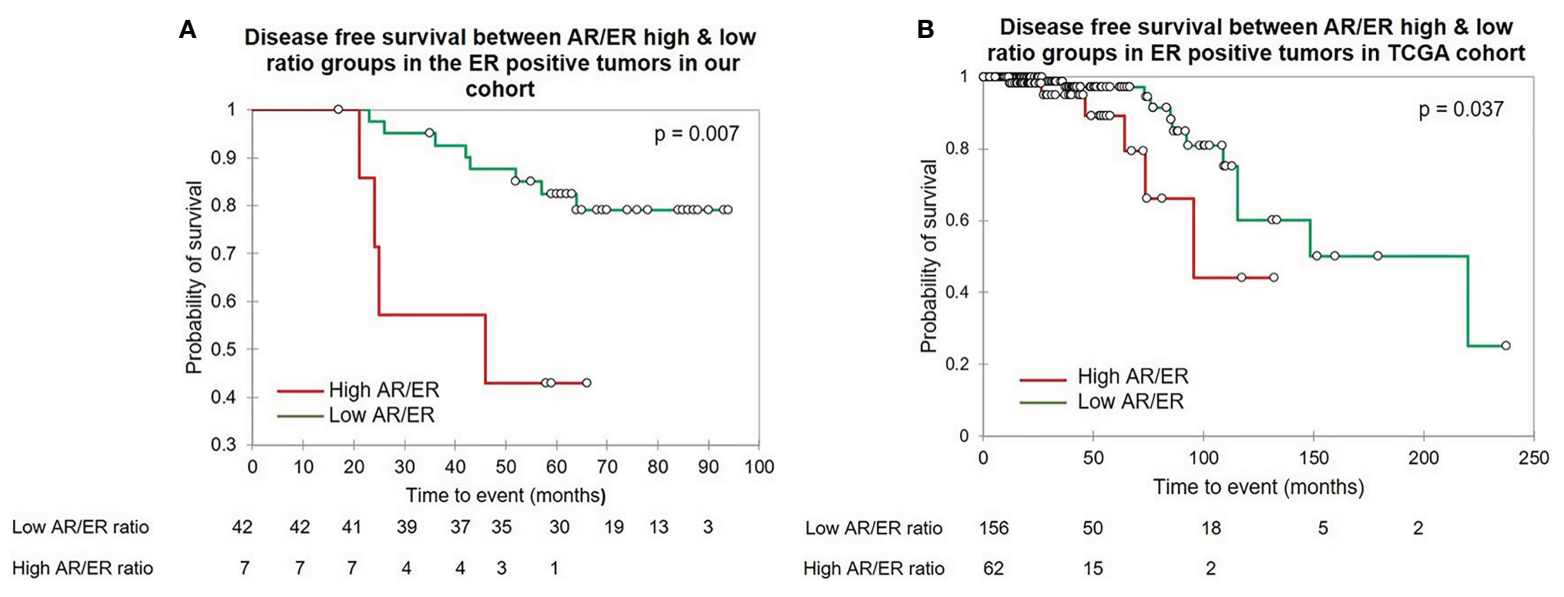
The Kaplan–Meier survival analysis in the ER positive patients ≤50 years of age. **(A)** The disease free survival between the high *vs* low AR/ER ratio groups in our cohort. **(B)** The disease free survival between the high *vs* low AR/ER ratio groups in the TCGA cohort.

Further, based on the ER protein expression by IHC, we divided the tumors into ER positive and negative and evaluated the prognostic significance of the AR/ER ratio independently within each category. Fifty-four (61%) of the 88 tumors were ER positive and women with tumors with higher ratio had significantly poorer survival when compared to the low ratio (mean survival time 41.8 *vs* 82.7 months, log rank test p = 0.007, **Figure 2A**). As observed in all patients ≤50 years age group, no difference in survival was seen with either ER transcript or AR transcript levels alone within the ER positive tumors. A similar analysis within the ER negative (by IHC) category did not show any difference in the survival between the AR/ER high and low ratio groups (mean survival time 68 *vs* 72.65 months, log rank test p = 0.68)

To investigate the prognostic significance of the AR/ER ratio in patients ≤50 years of age group, Cox proportional hazard analysis was performed with other known prognostic variables like tumor size, grade, and lymph node status. Univariate analysis showed (**Table 3**) prognostic significance of the high ratio with a hazard ratio of 2.6 (95% CI-1.0–6.5, p = 0.04) and 2.1 in multivariate analysis though not statistically significant (95% CI-0.8–5.8, p = 0.15). Similar analysis within ER positive tumors alone reconfirmed the prognostic relevance of the high AR/ER ratio with a significant hazard ratio of 4.6 (95% CI-1.35–15.37, p = 0.01) in univariate and 3.78 (95% CI-0.87–16.43, p=0.07) in multivariate analyses.

**Table 3:** Cox proportional hazard models of AR/ER ratio groups with other clinical variables in the patients ≤50 years in our cohort.

	Reference	Variable	Univariate (95% CI)				Multivariate (95% CI)			
			HR	Low	High	P-value	HR	Low	High	P-value
T-size	≤2 cm	>2 cm	0.7	0.2	1.7	0.38	0.7	0.2	2.1	0.51
LN status	Negative	Positive	1.6	0.6	4.1	0.38	1.6	0.5	4.8	0.43
Grade	Gr I & II	Gr III	1.6	0.58	4.08	0.37	0.6	0.06	5.5	0.65
Ratio groups	Low	High	2.6	1.0	6.5	0.04*	2.1	0.8	5.8	0.15
Treatment	HT	CT	0.61	0.12	3.16	0.56	0.18	0.03	1.06	0.06
		CT+HT	0.79	0.18	3.59	0.76	0.32	0.06	1.68	0.18

LN, lymphnode; HR, hazard ratio; HT, hormonal therapy; CT, chemotherapy. *p-value <0.05, statistically significant.

### External Validation in TCGA and METABRIC

To check if the results were recapitulated in other cohorts, we accessed the TCGA dataset (https://www.cancer.gov/tcga). This dataset had a total number of 1,082 breast cancer patients, of which 322 patients were ≤50 years. The AR/ER ratio of the transcripts of *AR and ESR1* was calculated and ranged from 0.02 to 4.07. A third quartile cut-off of the ratio at 0.88 was used to divide the tumors into high and low ratio groups. Comparison of clinical characters between the high and low ratio groups showed significantly different distribution of the ER status (p = 0.03) between the two groups, and no significant differences were observed in other features as shown in **Supplementary Table 2**. Kaplan–Meir survival analysis performed in the patients ≤50 years showed that the patients with high AR/ER ratio had a significantly poorer disease free survival than the low ratio tumors (mean survival time 74.8 *vs* 157.3 months, log rank test p = 0.003) similar to what we had seen in our cohort (**Supplementary Figure 1**). The Cox proportional hazard analysis showed prognostic significance of the high AR/ER ratio with a hazard ratio of 2.8 (95% CI-1.4–5.8, p = 0.005) in the univariate analysis and a significant hazard ratio of 3.2 (95% CI-1.4–7.3, p = 0.006) in the multivariate analysis (**Supplementary Table 3**).

Further, we performed similar analysis within ER positive tumors within TCGA; 218/322 were ER positive by IHC. Kaplan–Meir survival analysis in this subgroup showed patients with high AR/ER ratio had a significant poorer disease-free survival than the low ratio tumors (mean survival time 83 *vs* 163 months, log rank test p = 0.037), similar to our results (**Figure 2B**). Cox proportional hazard analysis showed prognostic significance of the high AR/ER ratio with a hazard ratio of 2.9 (95% CI-1.0–8.0, p = 0.046) in the univariate analysis and hazard ratio of 5.96 (95% CI-1.7–20.2, p = 0.004) in the multivariate analysis.

We also attempted to validate our results in the METABRIC dataset (22) (details in the **Supplementary Data**). As seen in our cohort, tumors with high ratio of AR/ER showed poorer survival than the low ratio tumors in both DFS (mean survival time 107.2 *vs* 142 months, log rank test p = 0.001) and BCCS (mean survival time 101.5 *vs* 137.5 months, log rank test p = 0.001) in the ≤50 years age group (**Supplementary Figures 2A, B**). Comparison between clinical variables between high and low ratio groups is shown in **Supplementary Table 4**. Cox proportional hazard analysis showed prognostic significance of the high AR/ER ratio with a hazard ratio of 1.8 (95% CI-1.25–2.52, p = 0.001) in the univariate analysis and hazard ratio of 1.2 (95% CI-0.82–1.78, p=0.33) in the multivariate analysis, though not statistically significant.

### Circulating Levels of Total Testosterone Are Higher in Patients ≤50 Years With High AR/ER Ratio

**Figure 3:**
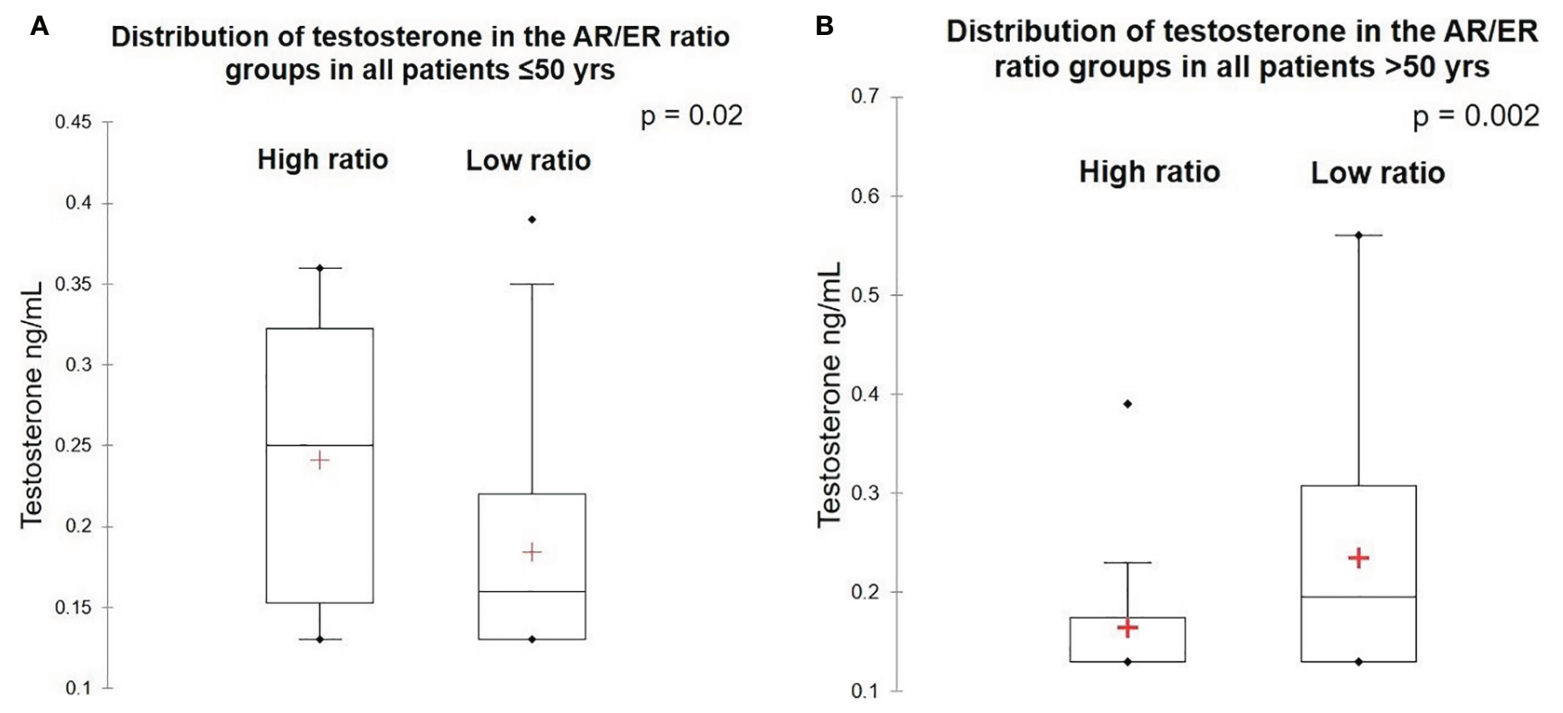
Levels of testosterone in the high and low AR/ER ratio groups. **(A)** Distribution of testosterone in the patients ≤50 years. **(B)** Distribution of testosterone in the patients >50 years.

To examine the association between circulating testosterone and AR expression in breast tumors, we estimated the total serum testosterone levels in patients at first diagnosis by chemiluminescence method. Among the total 270 patients, adequate serum was available in 169 patients (63 women were ≤50 years and 106 women were >50 years) for estimation of total testosterone level. The testosterone level ranged from 0.13 to 2.43 ng/ml with the mean value of 0.25 ng/m. No significant difference was observed in the circulating total testosterone levels between women with AR positive *versus* negative tumors (p = 0.847) and between patients ≤50 years and >50 years (p = 0.42). Women ≤50 years, with tumors having high AR/ER ratio tumors had significant high levels of testosterone compared to women with tumors with a low AR/ER ratio (p = 0.02). In contrast, high levels of circulating testosterone were observed in the patients >50 years with tumors having a low AR/ER ratio (p = 0.002) as shown in **Figures 3A, B**.

## Discussion

The prognostic value of AR expression in breast cancer has been evaluated in multiple breast cancer cohorts (14, 23–26). Steroid hormone nuclear receptors like estrogen and androgen receptors often crosstalk and influence the action of each other (13, 27). Evidence from *in vitro* studies suggests that AR competes to the same binding sites as ER leading to complex molecular mechanisms of their interaction (12). Despite the favorable prognostic role of AR in ER positive breast cancer, clinical studies have shown a subset of ER+AR+ tumors with a relative higher expression of AR compared to ER, often develops endocrine resistance when treated with tamoxifen (28).

Transcript levels of AR have been earlier used for its relevance as biomarker in clinical trial settings (29, 30). Higher levels of AR expression are seen in breast cancers of older women. Overall, we observed only 37% of all the tumors were positive for AR expression by IHC in the entire cohort. This proportion was not different in ≤50 years (34%). Only a minor proportion of ER positive tumors was AR positive (44%). Other studies from India have also reported similar proportions of AR positive tumors in their cohorts (31, 32). We have used the AR antibody clone AR441 and nuclear staining in >1% of the cells as positive similar to the guidelines for reporting on other nuclear receptors like ER and PR by immunohistochemical assay (33, 34). Higher proportions of AR positivity in other studies might be due to use of different antibody clones. Methodological differences observed in the published reports with use of more sensitive antibodies against AR and subjective interpretation of the AR expression by IHC with varying levels of cut-off, prompted us to use gene expression data for calculation of AR/ER ratio (35). Though moderate concordance was observed between the protein and mRNA of AR, estimation of transcript levels by q-PCR is more quantitative and permitted the estimation of the ratio in tumors which had ER expression below the threshold of detection for protein. In addition, we could also validate our results in a public dataset like TCGA and METABRIC which has limited data on protein expression.

Though ER positive breast cancers with AR expression tend to be well differentiated, Cochrane et al. reported about the prognostic significance of high AR/ER ratio with lower DFS and fourfold higher risk of failure during adjuvant tamoxifen treatment in ER positive breast cancers. Similarly, another study by Rangel et al. has shown that high AR/ER ratio is associated with aggressive features and is an independent indicator of worse prognosis in hormone receptor positive HER2 negative disease (15). Molecular subtyping of the tumors with high ratio in their study showed close to half of the tumors were intrinsically non-luminal though all were ER positive, and more than 60% of these tumors had either intermediate or high risk of recurrence by the PAM50/Prosigna assays. More recently, they further evaluated the gene expression of proliferation genes and showed tumors with high ratio were either luminal B or HER2 enriched with higher rate of proliferation and poor prognosis. Studies in both groups were limited to luminal tumors alone with median age group more than 60 years (18). Another study by Pizon et al. evaluated AR and ER in the circulating epithelial tumor cells (CETCs) in 66 BC tumors and found higher AR/ER ratio in patients with positive lymphnode and tamoxifen resistance (16). Our results are concordant with the findings from these studies in pre-menopausal women as well. Due to uncertainty in establishing menopausal status from medical records, we chose age 50 as a proxy for menopause, and pre-menopausal patients were defined as women younger than 50 years (as per the international average of natural menopause at 50 years, WHO).

Pre-menopausal women who have tumors with high AR/ER ratio had significantly high levels of circulating testosterone. Testosterone levels are more constant through the menstrual cycle, unlike estrogen and progesterone levels which are cyclical. Though multiple studies have shown the correlation of circulating testosterone with risk of developing breast cancer in post-menopausal women, relatively few studies have established the risk in pre-menopausal women (36). Previous studies in post-menopausal breast cancer women have shown significantly high levels of circulating testosterone than the normal controls (37) and further showed the association of high testosterone levels with worse prognosis in ER positive post-menopausal women (38). Regulation of AR depends on the hormonal milieu, and it is hypothesized that the discordance between AR and ER based signaling may be regulated by relative availability of each receptor (39). Testosterone is a precursor for estrogens and is converted by aromatase to either estradiol or 5*α*-dihydrotestosterone (DHT) by the enzyme 5*α* reductase in the tumor microenvironment (40). Our results of higher levels of total testosterone in tumors with high AR/ER ratio in ≤50 years group of tumors indicate these tumors are likely to be driven by the androgens (41). In contrast, higher levels of testosterone associated with lower levels of AR/ER ratio in >50 years tumor group may indicate their preferential conversion to estradiol leading to more ER driven tumors in the post-menopausal age group (42, 43). These results from predominantly pre-menopausal women with breast cancer suggest that it is not merely the presence or absence of AR expression but the relative activity with ER, as well as the hormonal milieu of the patient that determines clinical outcomes, indicating that both context and interactions ultimately influence tumor behavior.

Our study has several limitations. The major limitation is the small number of women under 50. Though our analysis replicated most of the findings in >50 years age group within our cohort, difference in DFS and BCCS between the high and low ratio groups did not reach statistical significance may be due to lack of long term follow-up (extending to median of 120 months or more) for development of endocrine resistance. We have not confined ourselves to ER positive, HER2 negative breast cancer alone as the prognostic utility of high AR/ER ratio is well established within this subtype. Lack of history on menstrual irregularities and information on BMI were other drawbacks due to which significance of higher levels of circulating steroids could not be evaluated further. Though we were able to replicate significance of AR/ER ratio in other external data sets, evaluation of circulating steroids cannot be validated in external data sets due to the absence of information on circulating steroids at the time of diagnosis and paucity of available cohorts with both serum and tissue for analysis associated with data on long term outcomes. These findings obviously need to be validated in larger cohorts along with standardized methods for detection of AR and its signaling.

## Data Availability Statement

The raw data supporting the conclusions of this article will be made available by the authors, without undue reservation.

## Ethics Statement

The studies involving human participants were reviewed and approved by the Institutional Ethical Committee, St John’s Medical College and Hospital, Bangalore and Sri Shankara Cancer Hospital and Research Centre, Bangalore. The patients/participants provided their written informed consent to participate in this study.

## Author Contributions

JSP: Analysis of data, conception and design of the study, performance of histological examination, and drafting the manuscript. SR: performance of experiments, analysis of data, and drafting the manuscript. AK: conception and design of the study. AA: patient consent and follow-up. AE: performance of IHC and sample collection. RR & SBS: surgical oncologist who enabled patient recruitment and tumor collection. TSS: conception and design of the study. All authors contributed to the article and approved the submitted version.

## Funding

This work was supported by DBT/Wellcome Trust India Alliance Fellowship/Grant [IA/CPHI/18/1/503938] awarded to JSP. Patient recruitment and follow-up activities was supported by philanthropic funding received from Nadathur Estates Private Ltd., Bangalore, India and from Bagaria Education Trust, Bangalore, India. The authors declare that this study received funding from Nadathur Estates Private Ltd., Bangalore, India and Bagaria Education Trust, Bangalore, India for patient recruitment and follow-up activities. The funder was not involved in the study design, collection, analysis, interpretation of data, the writing of this article or the decision to submit it for publication.

## Conflict of Interest

The authors declare that the research was conducted in the absence of any commercial or financial relationships that could be construed as a potential conflict of interest.

## Supplementary Material

Supplementary Figure 1 The Kaplan–Meier survival analysis in all patients ≤50 years of age for disease free survival (DFS) between the high and low AR/ER ratio groups in the TCGA cohort.

Supplementary Figure 2 The Kaplan–Meier survival analysis in METABRIC cohort in all the patients ≤50 years of age. **(A)** The disease-free survival between the high and low AR/ER ratio groups. **(B)** The breast cancer specific survival between the high and low AR/ER ratio groups.

## References

[1] Anders CK Fan C Parker JS Carey LA Blackwell KL Klauber-DeMore N. Breast Carcinomas Arising At a Young Age: Unique Biology or a Surrogate for Aggressive Intrinsic Subtypes? J Clin Oncol (2011) 29(1):e18–20. 10.1200/JCO.2010.28.9199 PMC3055864 21115855

[2] Sei HA Byung HS Seok WK Seung IK Jeong J Ko SS. Poor Outcome of Hormone Receptor-Positive Breast Cancer At Very Young Age Is Due to Tamoxifen Resistance: Nationwide Survival Data in Korea - A Report From the Korean Breast Cancer Society. J Clin Oncol (2007) 25:2360–8. 10.1200/JCO.2006.10.3754 17515570

[3] Min SY Kim Z Hur MH Yoon CS Park EH Jung KW. The Basic Facts of Korean Breast Cancer in 2013: Results of a Nationwide Survey and Breast Cancer Registry Database. J Breast Cancer (2016) 19:1–7. 10.4048/jbc.2016.19.1.1 27066090 PMC4822102

[4] Ma H Bernstein L Ross RK Ursin G. Hormone-Related Risk Factors for Breast Cancer in Women Under Age 50 Years by Estrogen and Progesterone Receptor Status: Results From a Case-Control and a Case-Case Comparison. Breast Cancer Res (2006) 66(8 Supplement):110. 10.1186/bcr1514 PMC1779482 16846528

[5] Liao S Hartmaier RJ McGuire KP Puhalla SL Luthra S Chandran UR. The Molecular Landscape of Premenopausal Breast Cancer. Breast Cancer Res (2015) 17:104. 10.1186/s13058-015-0618-8 26251034 PMC4531812

[6] Davis SR Wahlin-Jacobsen S. Testosterone in Women-the Clinical Significance. Lancet Diabetes Endocrinol (2015) 3:980–92. 10.1016/S2213-8587(15)00284-3 26358173

[7] Kaaks R Tikk K Sookthai D Schock H Johnson T Tjønneland A. Premenopausal Serum Sex Hormone Levels in Relation to Breast Cancer Risk, Overall and by Hormone Receptor Status-Results From the EPIC Cohort. Int J Cancer (2014) 134:1947–57. 10.1002/ijc.28528 24155248

[8] Fortner RT Eliassen AH Spiegelman D Willett WC Barbieri RL Hankinson SE. Premenopausal Endogenous Steroid Hormones and Breast Cancer Risk: Results From the Nurses’ Health Study II. Breast Cancer Res (2013) 15:R19. 10.1186/bcr3394 23497468 PMC3672790

[9] Folkerd E Dowsett M. Sex Hormones and Breast Cancer Risk and Prognosis. Breast (2013) 22:S38–43. 10.1016/j.breast.2013.07.007 24074790

[10] Zhao S Chlebowski RT Anderson GL Kuller LH Manson JAE Gass M. Sex Hormone Associations With Breast Cancer Risk and the Mediation of Randomized Trial Postmenopausal Hormone Therapy Effects. Breast Cancer Res (2014) 16(2):R30. 10.1186/bcr3632 24670297 PMC4053241

[11] Kensler KH Regan MM Heng YJ Baker GM Pyle ME Schnitt SJ. Prognostic and Predictive Value of Androgen Receptor Expression in Postmenopausal Women With Estrogen Receptor-Positive Breast Cancer: Results From the Breast International Group Trial 1-98. Breast Cancer Res (2019) 21:1–11. 10.1186/s13058-019-1118-z 30795773 PMC6387478

[12] McNamara KM Moore NL Hickey TE Sasano H Tilley WD. Complexities of Androgen Receptor Signalling in Breast Cancer. Endocr Relat Cancer (2014) 21(4):T161–81. 10.1530/ERC-14-0243 24951107

[13] D’Amato NC Gordon MA Babbs B Spoelstra NS Butterfield KTC Torkko KC. Cooperative Dynamics of AR and ER Activity in Breast Cancer. Mol Cancer Res (2016) 14:1054–67. 10.1158/1541-7786.MCR-16-0167 PMC5107172 27565181

[14] Cochrane DR Bernales S Jacobsen BM Cittelly DM Howe EN D’Amato NC. Role of the Androgen Receptor in Breast Cancer and Preclinical Analysis of Enzalutamide. Breast Cancer Res (2014) 16:1–19. 10.1186/bcr3599 PMC3978822 24451109

[15] Rangel N Rondon-Lagos M Annaratone L Osella-Abate S Metovic J Mano MP. The Role of the AR/ER Ratio in ER-Positive Breast Cancer Patients. Endocr Relat Cancer (2018) 25:163–72. 10.1530/ERC-17-0417 29386247

[16] Pizon M Lux D Pachmann U Pachmann K Schott D. Influence of Endocrine Therapy on the Ratio of Androgen Receptor (AR) to Estrogen Receptor (ER) Positive Circulating Epithelial Tumor Cells (Cetcs) in Breast Cancer. J Transl Med (2018) 16(1):356. 10.1186/s12967-018-1724-z 30547831 PMC6295012

[17] Bronte G Rocca A Ravaioli S Scarpi E Bonafè M Puccetti M. Evaluation of Androgen Receptor in Relation to Estrogen Receptor (AR/ER) and Progesterone Receptor (AR/Pgr): A New Must in Breast Cancer? J Oncol (2019) 2019:1–6. 10.1155/2019/1393505 PMC6487115 31110518

[18] Rangel N Rondon-Lagos M Annaratone L Aristizábal-Pachon AF Cassoni P Sapino A. Ar/Er Ratio Correlates With Expression of Proliferation Markers and With Distinct Subset of Breast Tumors. Cells (2020) 9(4):1064. 10.3390/cells9041064 PMC7226480 32344660

[19] Mousavi-Jarrrahi SH Kasaeian A Mansori K Ranjbaran M Khodadost M Mosavi-Jarrahi A. Addressing the Younger Age At Onset in Breast Cancer Patients in Asia: An Age-Period-Cohort Analysis of Fifty Years of Quality Data From the International Agency for Research on Cancer. ISRN Oncol (2013) 2013:1–8. 10.1155/2013/429862 PMC3786111 24102030

[20] Chaturvedi M Vaitheeswaran K Satishkumar K Das P Stephen S Nandakumar A. Time Trends in Breast Cancer Among Indian Women Population: An Analysis of Population Based Cancer Registry Data. Indian J Surg Oncol (2015) 6:427–34. 10.1007/s13193-015-0467-z PMC4809853 27065669

[21] Korlimarla A Prabhu JS Anupama CE Remacle J Wahi K Sridhar TS. Separate Quality-Control Measures Are Necessary for Estimation of RNA and Methylated DNA From Formalin-Fixed, Paraffin-Embedded Specimens by Quantitative PCR. J Mol Diagn (2014) 16:253–60. 10.1016/j.jmoldx.2013.11.003 24412525

[22] Curtis C Shah SP Chin SF Turashvili G Rueda OM Dunning MJ. The Genomic and Transcriptomic Architecture of 2,000 Breast Tumours Reveals Novel Subgroups. Nature (2012) 486:346–52. 10.1038/nature10983 PMC3440846 22522925

[23] Feng J Li L Zhang N Liu J Zhang L Gao H. Androgen and AR Contribute to Breast Cancer Development and Metastasis: An Insight of Mechanisms. Oncogene (2017) 36:2775–90. 10.1038/onc.2016.432 27893717

[24] Giovannelli P Di Donato M Galasso G Di Zazzo E Bilancio A Migliaccio A. The Androgen Receptor in Breast Cancer. Front Endocrinol (Lausanne) (2018) 9:492. 10.3389/fendo.2018.00492 30210453 PMC6122126

[25] Michmerhuizen AR Spratt DE Pierce LJ Speers CW. Are We There Yet? Understanding Androgen Receptor Signaling in Breast Cancer. NPJ Breast Cancer (2020) 6:1–19. 10.1038/s41523-020-00190-9 33062889 PMC7519666

[26] Hickey TE Robinson JLL Carroll JS Tilley WD. Minireview: The Androgen Receptor in Breast Tissues: Growth Inhibitor, Tumor Suppressor, Oncogene? Mol Endocrinol (2012) 26:1252–67. 10.1210/me.2012-1107 PMC3404296 22745190

[27] Karamouzis MV Papavassiliou KA Adamopoulos C Papavassiliou AG. Targeting Androgen/Estrogen Receptors Crosstalk in Cancer. Trends Cancer (2016) 2:35–48. 10.1016/j.trecan.2015.12.001 28741499

[28] Basile D Cinausero M Iacono D Pelizzari G Bonotto M Vitale MG. Androgen Receptor in Estrogen Receptor Positive Breast Cancer: Beyond Expression. Cancer Treat Rev (2017) 61:15–22. 10.1016/j.ctrv.2017.09.006 29078133

[29] Bozovic-Spasojevic I Zardavas D Brohee S Ameye L Fumagalli D Ades F. The Prognostic Role of Androgen Receptor in Patients With Early-Stage Breast Cancer: A Metaanalysis of Clinical and Gene Expression Data. Clin Cancer Res (2017) 23:2702–12. 10.1158/1078-0432.CCR-16-0979 28151718

[30] Barton VN Gordon MA Richer JK Elias A. Anti-Androgen Therapy in Triple-Negative Breast Cancer. Ther Adv Med Oncol (2016) 8:305–8. 10.1177/1758834016646735 PMC4952024 27482289

[31] Vellaisamy G Tirumalae R Inchara Y. Expression of Androgen Receptor in Primary Breast Carcinoma and Its Relation With Clinicopathologic Features, Estrogen, Progesterone, and Her-2 Receptor Status. J Cancer Res Ther (2019) 15:989–93. 10.4103/jcrt.JCRT_572_17 31603099

[32] Anand A Singh KR Kumar S Husain N Kushwaha JK Sonkar AA. Androgen Receptor Expression in an Indian Breast Cancer Cohort With Relation to Molecular Subtypes and Response to Neoadjuvant Chemotherapy - A Prospective Clinical Study. Breast Care (2017) 12:160–4. 10.1159/000458433 PMC5527170 28785183

[33] Allison KH Hammond MEH Dowsett M McKernin SE Carey LA Fitzgibbons PL. Estrogen and Progesterone Receptor Testing in Breast Cancer: ASCO/CAP Guideline Update. J Clin Oncol (2020) 38:1346–66. 10.1200/JCO.19.02309 31928404

[34] Park S Koo J Park HS Kim JH Choi SY Lee JH. Expression of Androgen Receptors in Primary Breast Cancer. Ann Oncol (2009) 21:488–92. 10.1093/annonc/mdp510 19887463

[35] Nour El Hoda SI Khairy RA Talaat SM Abd El-Fattah FA. Immunohistochemical Expression of Androgen Receptors (AR) in Various Breast Cancer Subtypes. Open Access Maced J Med Sci (2019) 7:1259–65. 10.3889/oamjms.2019.311 PMC6514328 31110566

[36] Dorgan JF Stanczyk FZ Kahle LL Brinton LA. Prospective Case-Control Study of Premenopausal Serum Estradiol and Testosterone Levels and Breast Cancer Risk. Breast Cancer Res (2010) 12:R98. 10.1186/bcr2779 21087481 PMC3046441

[37] Secreto G Zumoff B. Role of Androgen Excess in the Development of Estrogen Receptor-Positive and Estrogen Receptor-Negative Breast Cancer. Anticancer Res (2012) 32:3223–8. 10.1186/1471-2407-12-599 22843896

[38] Venturelli E Orenti A Fabricio ASC Garrone G Agresti R Paolini B. Observational Study on the Prognostic Value of Testosterone and Adiposity in Postmenopausal Estrogen Receptor Positive Breast Cancer Patients. BMC Cancer (2018) 18:651. 10.1186/s12885-018-4558-4 29895278 PMC5998599

[39] Creevey L Bleach R Madden SF Toomey S Bane FT Vareslija D. Altered Steroid Milieu in AI-Resistant Breast Cancer Facilitates AR Mediated Gene-Expression Associated With Poor Response to Therapy. Mol Cancer Ther (2019) 18:1731–43. 10.1158/1535-7163.MCT-18-0791 31289138

[40] McNamara KM Sasano H. The Intracrinology of Breast Cancer. J Steroid Biochem Mol Biol (2015) 145:172–8. 10.1016/j.jsbmb.2014.04.004 24751707

[41] Secreto G Girombelli A Krogh V. Androgen Excess in Breast Cancer Development: Implications for Prevention and Treatment. Endocr Relat Cancer (2019) 26:R81–94. 10.1530/ERC-18-0429 30403656

[42] Sikora MJ Cordero KE Larios JM Johnson MD Lippman ME Rae JM. The Androgen Metabolite 5α-Androstane-3β,17β-Diol (3βadiol) Induces Breast Cancer Growth Via Estrogen Receptor: Implications for Aromatase Inhibitor Resistance. Breast Cancer Res Treat (2009) 115:289–96. 10.1007/s10549-008-0080-8 PMC2728015 18521740

[43] Hanamura T Niwa T Nishikawa S Konno H Gohno T Tazawa C. Androgen Metabolite-Dependent Growth of Hormone Receptor-Positive Breast Cancer as a Possible Aromatase Inhibitor-Resistance Mechanism. Breast Cancer Res Treat (2013) 139:731–40. 10.1007/s10549-013-2595-x 23780684

