# Supplementary figures and images for: Pre-Menopausal Women With Breast Cancers Having High AR/ER Ratios in the Context of Higher Circulating Testosterone Tend to Have Poorer Outcomes
Source: Front Endocrinol (Lausanne). 2021 Jun 21;12:679756. doi: 10.3389/fendo.2021.679756 (PMC8256854; doi:10.3389/fendo.2021.679756)

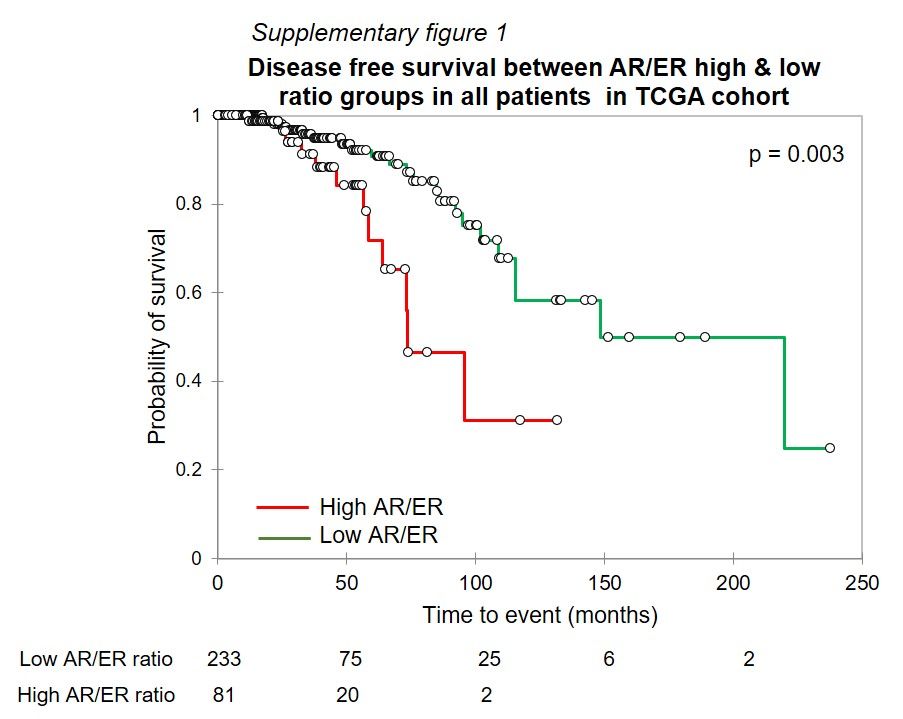

Supplement: Supplementary Figure 1 — The Kaplan–Meier survival analysis in all patients ≤50 years of age for disease free survival (DFS) between the high and low AR/ER ratio groups in the TCGA cohort. [file Image_1.jpg]

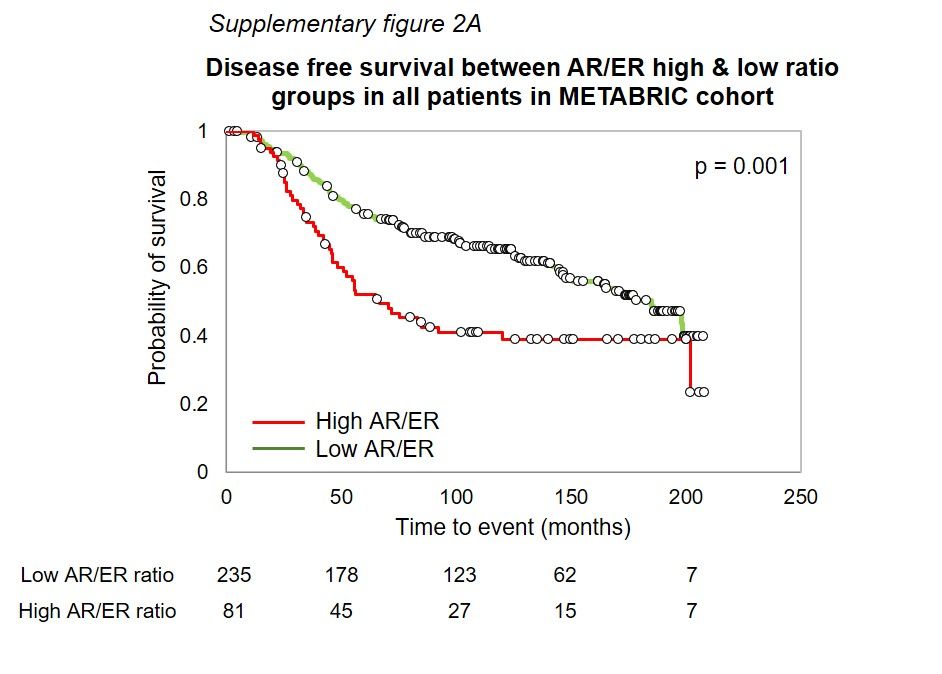

Supplement: Supplementary Figure 2 — The Kaplan–Meier survival analysis in METABRIC cohort in all the patients ≤50 years of age. (A) The disease-free survival between the high and low AR/ER ratio groups. (B) The breast cancer specific survival between the high and low AR/ER ratio groups. [file Image_2.jpg]

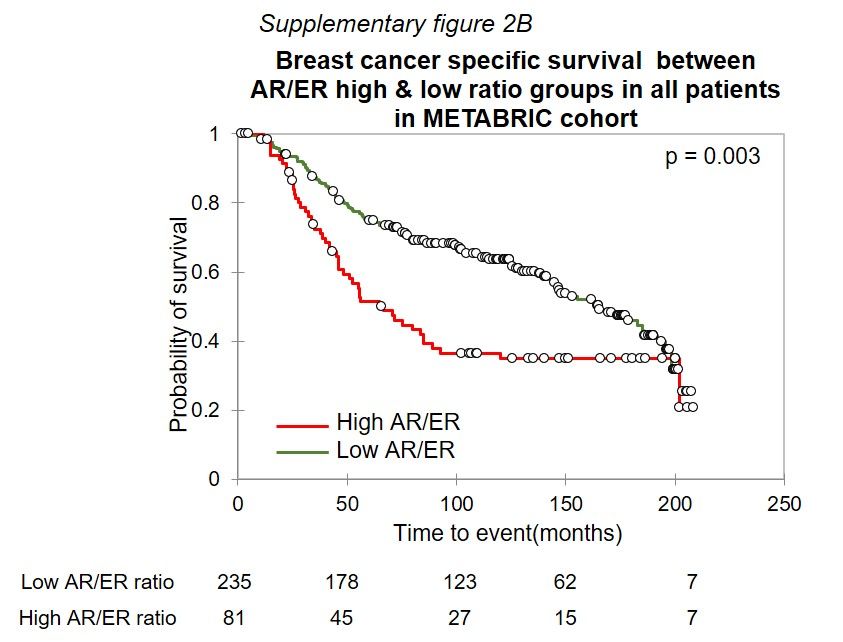

Supplement: Supplementary file 3 [file Image_3.jpg]
