# Supplementary material for: Pre-Menopausal Women With Breast Cancers Having High AR/ER Ratios in the Context of Higher Circulating Testosterone Tend to Have Poorer Outcomes
Source: Front Endocrinol (Lausanne). 2021 Jun 21;12:679756. doi: 10.3389/fendo.2021.679756 (PMC8256854; doi:10.3389/fendo.2021.679756)
Supplement: Supplementary file 4 [file Table_1.docx]

**Supplementary Table1**: Primer sequences for *AR* and *ESR1* transcripts

| **Gene** | **Primer Sequence** | **Product Size** |
| --- | --- | --- |
| *AR* | F-TGGGCTGGCGATCCTTC | 93 |
|  | R-TCCGGGACTTGTGCATGCGG |  |
| *ESR1* | F-GCAGGGAGAGGAGTTTGT | 65 |
|  | R-GACTTCAGGGTGCTGGAC |  |
