# Supplementary material for: Pre-Menopausal Women With Breast Cancers Having High AR/ER Ratios in the Context of Higher Circulating Testosterone Tend to Have Poorer Outcomes
Source: Front Endocrinol (Lausanne). 2021 Jun 21;12:679756. doi: 10.3389/fendo.2021.679756 (PMC8256854; doi:10.3389/fendo.2021.679756)
Supplement: Supplementary file 5 [file Table_2.docx]

**Supplementary Table 2**: Comparison of clinicopathological features between the AR/ER

ratio groups in TCGA cohort in patients ≤50 yrs of age

| **Clinicopathological**  **characteristics** |  | **High AR/ER ratio**  **(N=81)** | **Low AR/ER ratio**  **(N=237)** | **p-value** |
| --- | --- | --- | --- | --- |
|  |  | N (%) | N (%) |  |
| Age | Median | 45 | 45 |  |
| Lymph Node | Positive | 34(44) | 124(53) | 0.4 |
|  | Negative | 41(53) | 107(46) |  |
|  | Nx | 2(3) | 4(1) |  |
|  | NA | 4 | 2 |  |
| Stage | I | 12(16) | 43(19) | 0.88 |
|  | II | 47(62) | 141(61) |  |
|  | III | 16(21) | 43(18) |  |
|  | IV | 1(1) | 5(2) |  |
|  | NA | 5 | 5 |  |
| T-size | 1 | 17(22) | 64(27) | 0.57 |
|  | 2 | 47(61) | 132(56) |  |
|  | 3 | 12(16) | 34(15) |  |
|  | 4 | 0 | 4(2) |  |
|  | Tx | 1(1) | 1(1) |  |
|  | NA | 4 | 2 |  |
| Estrogen Receptor | Positive | 62(83) | 158(71) | 0.03* |
|  | Negative | 13(17) | 66(30) |  |
|  | NA | 6 | 13 |  |
| Progesterone Receptor | Positive | 56(74) | 142(64) | 0.11 |
|  | Negative | 20(26) | 81(36) |  |
|  | NA | 5 | 14 |  |
| HER2 | Positive | 21(30) | 25(13) | 0.005* |
|  | Negative | 40(57) | 125(67) |  |
|  | Equivocal | 9(12) | 38(20) |  |
|  | NA | 11 | 49 |  |

*p-value <0.05, statistically significant
