# Supplementary material for: Pre-Menopausal Women With Breast Cancers Having High AR/ER Ratios in the Context of Higher Circulating Testosterone Tend to Have Poorer Outcomes
Source: Front Endocrinol (Lausanne). 2021 Jun 21;12:679756. doi: 10.3389/fendo.2021.679756 (PMC8256854; doi:10.3389/fendo.2021.679756)
Supplement: Supplementary file 6 [file Table_3.docx]

**Supplementary Table 3 :** Cox proportional hazard models of AR/ER ratio groups with other clinical variables in the TCGA cohort in the patients ≤50 years of age (N=322)

|  |  |  | Univariate (95% CI) | | | | Multivariate (95% CI) | | | |
| --- | --- | --- | --- | --- | --- | --- | --- | --- | --- | --- |
|  | Reference | Variable | HR | Low | High | P-value | HR | Low | High | P-value |
| T-size | T1 | T2, T3, T4 | 1.7 | 0.7 | 3.9 | 0.21 | 1.9 | 0.8 | 4.6 | 0.12 |
| LN status | Negative | Positive | 0.7 | 0.4 | 1.4 | 0.34 | 0.6 | 0.3 | 1.2 | 0.13 |
| Ratio groups | Low | High | 2.8 | 1.4 | 5.8 | 0.005* | 3.2 | 1.4 | 7.3 | 0.006* |

HR : Hazard ratio ,LN : Lymphnode ,*p-value <0.05, statistically significant
