# Supplementary material for: Pre-Menopausal Women With Breast Cancers Having High AR/ER Ratios in the Context of Higher Circulating Testosterone Tend to Have Poorer Outcomes
Source: Front Endocrinol (Lausanne). 2021 Jun 21;12:679756. doi: 10.3389/fendo.2021.679756 (PMC8256854; doi:10.3389/fendo.2021.679756)
Supplement: Supplementary file 7 [file Table_4.docx]

**Supplementary Table 4**: Comparison of clinicopathological features between the AR/ER ratio groups in METABRIC cohort in the patients ≤50 years

| **Clinicopathological**  **characteristics** |  | **High AR/ER ratio**  **(N=81)** | **Low AR/ER ratio**  **(N=235)** | **p-value** |
| --- | --- | --- | --- | --- |
|  |  | N (%) | N (%) |  |
| Age | Median | 41.9 | 43.7 |  |
| T size | Median | 2.5 | 2.1 |  |
| Lymph Node | Positive | 59(73) | 113(48) | <0.0001* |
|  | Negative | 22(27) | 122(51) |  |
| Stage | I | 10(17) | 64(34) | 0.032* |
|  | II | 35(62) | 101(54) |  |
|  | III | 10(17) | 18(10) |  |
|  | IV | 0 | 1(0) |  |
|  | Not Available | 25 | 51 |  |
| Grade | I | 1(1) | 17(7) | <0.0001* |
|  | II | 7(9) | 88(38) |  |
|  | III | 71(88) | 126(55) |  |
|  | Not Available | 2 | 4 |  |
| Estrogen Receptor | Positive | 2(2) | 174(74) | <0.0001* |
|  | Negative | 79(98) | 61(25) |  |
| Progesterone Receptor | Positive | 6(7) | 145(62) | <0.0001* |
|  | Negative | 75(93) | 90(38) |  |
| HER2 | Positive | 45(56) | 23(10) | <0.0001* |
|  | Negative | 36(44) | 212(90) |  |

*p-value <0.05, statistically significant
