# Supplementary material for: Pre-Menopausal Women With Breast Cancers Having High AR/ER Ratios in the Context of Higher Circulating Testosterone Tend to Have Poorer Outcomes
Source: Front Endocrinol (Lausanne). 2021 Jun 21;12:679756. doi: 10.3389/fendo.2021.679756 (PMC8256854; doi:10.3389/fendo.2021.679756)
Supplement: Supplementary file 8 [file DataSheet_1.docx]

**Supplemenaty data : Validation in METABRIC:** We accessed data from 316 cases of breast cancer ≤50 years from a total of 1905 breast cancer cases available. Ratio of AR/ER was calculated using the transcript levels of *AR* and *ESR1* which ranged from 0.55 to 1.69 with a third quartile value of 0.99. Tumors were therefore divided into low and high ratio groups using a cut off of 1.0 and the DFS and BCSS were calculated using Kaplan Meier survival analysis. As seen in our cohort, tumors with high ratio of AR/ER showed poorer survival than the low ratio tumors in both DFS (mean survival time 107.2 months Vs 142 months, log rank test p=0.001) and BCCS (mean survival time 101.5 months Vs 137.5 months , log rank test p=0.001) in the ≤50 years age group (Supplementary figure 2A and 2B).Comparison of clinical variables between high and low ratio groups showed higher proportion of lymph node positive (p<0.0001), high grade (p<0.0001) and ER negative tumors(p<0.0001) in the high ratio group as seen in the Supplementary table 4. Cox proportional hazard analysis showed prognostic significance of the high AR/ER ratio with a hazard ratio of 1.8 (95% CI-1.25 - 2.52,p=0.001) in the univariate analysis and hazard ratio of 1.2 (95% CI-0.82 - 1.78, p=0.33) in the multivariate analysis, though not statistically significant. Of the 316 patients ≤50 years ,176 tumors were ER positive and only 2/176 (1.1%) tumors belonged to the high ratio group. Hence, further validation of the ratio within ER positive tumors could not be attempted.
